# Visual Nanoprobe-Enhanced Loop-Mediated Isothermal Amplification Protocol for Rapid Detection of Infectious Laryngotracheitis Virus from Avian Respiratory Swabs

**DOI:** 10.21769/BioProtoc.5595

**Published:** 2026-02-05

**Authors:** Pablo Cea-Callejo, Claudia Trenado, Ana Doménech, Ricardo Madrid, Laura Benítez

**Affiliations:** 1 Department of Genetics, Physiology, and Microbiology, Faculty of Biological Sciences, Complutense University of; Madrid (UCM), Madrid, Spain; 2 Department of Animal Health, Veterinary Faculty, Complutense University of Madrid (UCM), Madrid, Spain; 3 Research Group of “Animal Viruses”, Complutense University of Madrid, Madrid, Spain

**Keywords:** Infectious laryngotracheitis virus (ILTV), Loop-mediated isothermal amplification (LAMP), DNA nanoprobes, Gold nanoparticles (AuNPs), Point-of-care test (POCT)

## Abstract

A prompt and accurate diagnosis of respiratory viral diseases in intensive poultry production is essential to safeguard animal health and ensure the economic sustainability of farms. Currently, much effort is being devoted to preventing the spread of the avian influenza virus in farms. However, the diagnosis of other relevant respiratory viruses, as infectious laryngotracheitis virus (ILTV), is also crucial. Indeed, infection by ILTV does lead to substantial economic losses due to high morbidity, reduced growth, and decreased productivity, making rapid detection a critical aspect of disease control. Conventional diagnostics, including PCR and qPCR, while sensitive and specific, require expensive laboratory infrastructure and well-trained personnel, limiting their deployment in field settings where immediate intervention is most valuable. To address these limitations, this protocol describes a portable molecular diagnostic workflow based on loop-mediated isothermal amplification (LAMP) combined with gold nanoparticle–DNA nanoprobes for specific and visual detection of ILTV directly at the point of need. Gold nanoparticles synthesized via the Turkevich method are functionalized with thiolated DNA probes, which undergo full-length, sequence-specific hybridization to LAMP amplicons, enabling a naked-eye colorimetric readout. The procedure integrates streamlined steps for DNA probe preparation, nanoparticle synthesis and assembly, and minimal sample processing, compatible with on-farm deployment. Results obtained with this workflow on field samples demonstrated 100% sensitivity and specificity, matching the performance of gold-standard assays. This approach offers a rapid, cost-effective, and equipment-free detection system of viral pathogens, enabling timely decision-making for disease containment and biosecurity. By overcoming the barriers of conventional diagnostics, this protocol enables producers with powerful tools for efficient monitoring and response to respiratory outbreaks in poultry farms.

Key features

• Direct ILTV detection in respiratory swabs in 35–45 min, bypassing DNA extraction with a rapid viral lysis step.

• Specific colorimetric readout via DNA nanoprobes with visual interpretation, requiring no specialized equipment or lab infrastructure.

• Achieves 100% sensitivity and specificity compared to qPCR, with a detection limit of 200 viral copies per reaction; validated in lab conditions with field samples.

• Modular design: Enables multiplex and customizable detection of other poultry pathogens, supporting rapid kit development and broad field application.

## Graphical overview


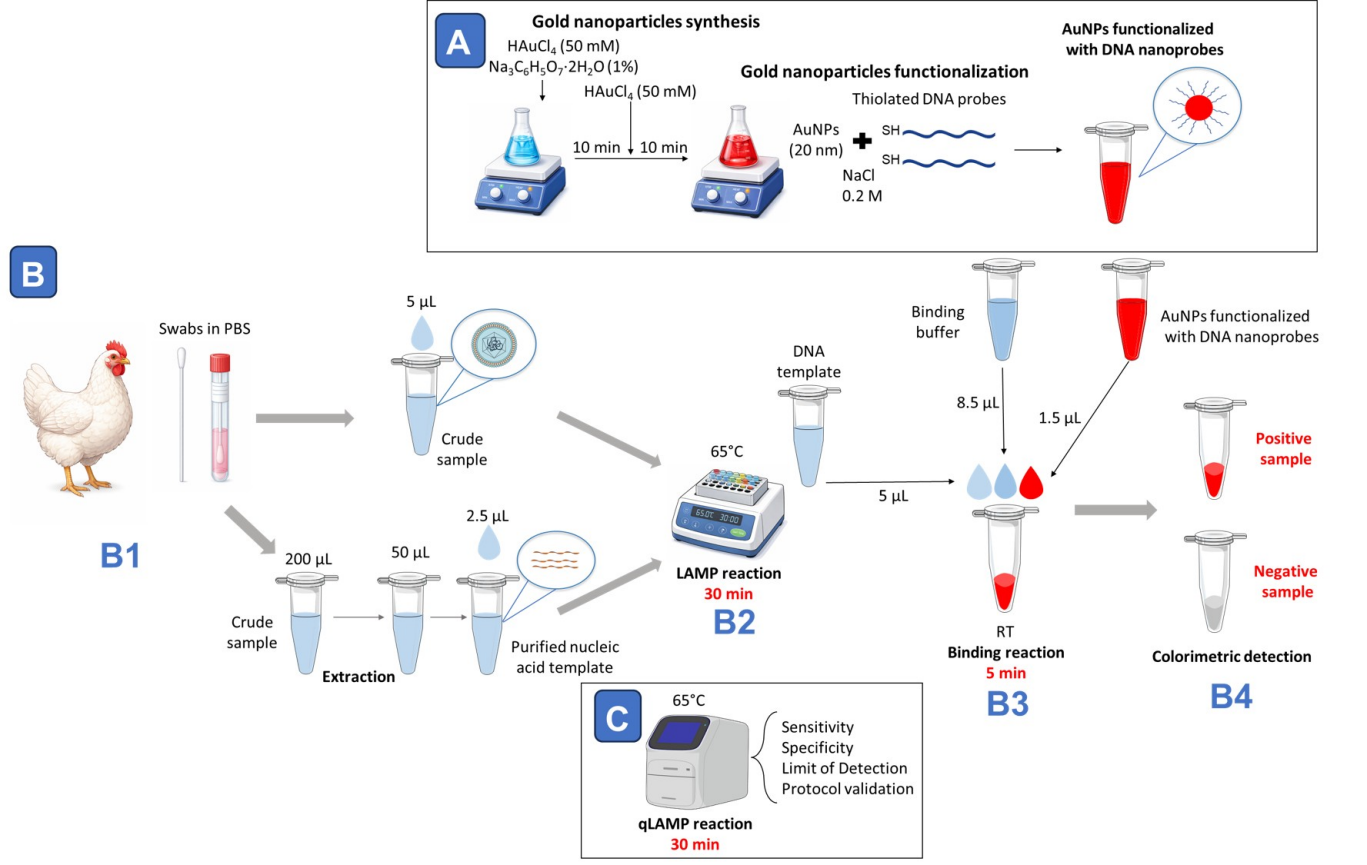


**Workflow overview for avian respiratory virus detection combining loop-mediated isothermal amplification (LAMP) with AuNP-nanoprobe-based colorimetric readout.** (A) For DNA-nanoprobe preparation, gold nanoparticles with a diameter of approximately 20 nm are synthesized using a two-step citrate reduction method and subsequently functionalized with thiolated DNA probes in the presence of NaCl. (B1) Oropharyngeal swabs collected in PBS are processed either as crude samples or following nucleic acid extraction. (B2) Samples are subjected to a 30-min LAMP reaction at 65 °C. (B3) After amplification, 5 μL of LAMP product is mixed with binding buffer (8.5 μL) and DNA nanoprobes (1.5 μL) and incubated at room temperature for 5 min. (B4) Hybridization with target DNA stabilizes nanoprobes in positive samples; in negative samples, absence of detection leads to nanoparticle destabilization and reaction clearing, enabling visual discrimination. (C) In parallel, qLAMP fluorescence reactions performed at 65 °C for 30 min are used to evaluate assay sensitivity, specificity, limit of detection, and overall protocol validation.

## Background

Infectious laryngotracheitis (ILT) is a highly contagious respiratory disease that affects chickens, posing a significant economic threat to poultry production worldwide due to severe outbreaks characterized by high morbidity and variable mortality [1]. The etiological agent, infectious laryngotracheitis virus (ILTV), is a herpesvirus classified as *Iltovirus gallidalpha 1*, belonging to the *Alphaherpesvirinae* subfamily, with a double-stranded DNA genome of approximately 150 kb encoding around 80 predicted viral proteins. ILT manifests clinically in acute or subacute forms, with mortality rates up to 50% and characteristic symptoms including conjunctivitis, nasal discharge, respiratory distress, decreased egg production, and weight loss. These signs overlap with other avian respiratory infections such as infectious bronchitis virus (IBV) and avian metapneumovirus (aMPV), complicating diagnosis [2,3]. Additionally, ILTV establishes lifelong latency in trigeminal ganglia, rendering infected birds lifelong carriers and recurrent sources of infection, which further challenges control efforts in poultry farms.

Transmission occurs mainly through direct contact, and intensive farming conditions facilitate rapid spread. Early and accurate diagnosis of ILTV is crucial for timely intervention and outbreak containment to reduce economic losses in poultry production. Conventional diagnostic approaches have evolved from laborious and time-consuming laboratory-based methods to molecular detection techniques that include conventional PCR and real-time quantitative PCR (qPCR) [4]. Although highly sensitive and specific, these molecular methods require specialized equipment, well-trained personnel, and strict sample transport conditions, which limit their applicability for on-site, rapid diagnosis in field or low-resource settings [5].

Loop-mediated isothermal amplification (LAMP) has emerged as a promising alternative for point-of-care testing (POCT) due to its rapid amplification of nucleic acids at a constant temperature without the need for thermal cycling and its robustness against inhibitors commonly found in clinical samples. The LAMP reaction employs six primers recognizing eight distinct regions of the target DNA, enhancing specificity and amplification efficiency, which results in rapid and high-yield DNA amplification within less than 1 h [6]. Despite these advantages, visualizing LAMP amplicons reliably and specifically remains a challenge, as traditional methods such as pH indicators or turbidity assays can produce false positives due to nonspecific amplification [7].

To overcome these limitations, this study describes a novel diagnostic system that integrates LAMP targeting the ILTV glycoprotein E gene with a specific colorimetric detection method using DNA nanoprobes. The unique plasmonic properties of gold nanoparticles (AuNPs) enable naked-eye detection based on color change maintained by specific DNA–DNA hybridization, providing a highly specific and easy readout without complex fluorescence detection instruments or sophisticated laboratory infrastructure. The assay is compatible with both extracted nucleic acids and crude respiratory swab samples, the latter enabled by heat-induced lysis that eliminates the need for prior extraction and shortens the diagnostic turnaround to 35–45 min. The method achieves a detection limit of 200 viral copies per reaction, demonstrating 100% sensitivity and specificity compared to qPCR assays [8].

Moreover, the protocol is fully scalable and adaptable for development into diagnostic kits suitable for widespread use. Its modular primer design enables easy customization for detecting other poultry viral pathogens with minimal assay re-optimization. Furthermore, this platform supports multiplex detection, allowing simultaneous identification of multiple viral pathogens in a single reaction tube, facilitating comprehensive field monitoring with streamlined workflows and rapid visual readouts.

In summary, this LAMP-nanoprobe system represents an advance in rapid, cost-effective, point-of-care diagnostics for ILTV and other infectious agents, enhancing timely disease management and biosecurity in poultry production worldwide.

## Materials and reagents

**Biological materials**

1. Oropharyngeal swabs from chickens and turkeys positive for infectious laryngotracheitis virus (ILTV) resuspended in 1 mL of 1× PBS (crude samples), kindly provided by the Centro de Sanidad Avícola de Cataluña y Aragón (CESAC)

**Reagents**

**For sample collection, nucleic acid extraction, and LAMP amplification/visualization:**

1. Swabs for oropharyngeal sample collection (Merck, catalog number: WHAWB100032)

2. MagMAX Viral/Pathogen I Nucleic Acid Isolation kit (Invitrogen, catalog number: A48310)

3. DNA Zap for degradation solution (Invitrogen, catalog number: AM9890)

4. WarmStart^®^ LAMP kit (DNA & RNA) (New England Biolabs, catalog number: E1700)

5. LAMP fluorescent dye (New England Biolabs, catalog number: B1700)

6. Nuclease-free water (Invitrogen, catalog number: 10977023)

7. Primers targeting ILTV glycoprotein E (*gE*) gene (Macrogen-Inc., South Korea, custom synthesis)

8. Agarose (Bio-Rad Laboratories, catalog number: 4561094)

9. TAE 50× for molecular biology (Condalab, catalog number: A4686-1000)

10. GelRed^TM^ nucleic acid gel stain (Biotium Inc., catalog number: 41003)

11. 6× DNA loading buffer (TaKaRa, catalog number: 9156)

12. NZYDNA ladder VI, 50–1,500 bp (NZYtech, catalog number: MB08903)

**For AuNPs synthesis:**

1. Chloroauric acid (HAuCl_4_·3H_2_O) (Sigma-Aldrich, catalog number: 254169)

2. Trisodium citrate dihydrate (Na_3_C_6_H_5_O_7_·2H_2_O) (Sigma-Aldrich, catalog number: 71370)

**For AuNPs functionalization:**

1. Sodium chloride (NaCl) (Sigma-Aldrich, catalog number: 7647-14-5)

2. PBS 1× (Condalab, catalog number: 4015)

3. DNA oligonucleotide nanoprobes (Macrogen-Inc., South Korea, customized synthesis, 5’-thiol modification for nanoprobe functionalization)

4. Magnesium chloride (MgCl_2_), 100 mM solution (Sigma-Aldrich, catalog number: 208337)

5. Tween-20 (polyoxyethylene sorbitan monolaurate) (Sigma-Aldrich, catalog number: P9416)

6. Sodium acetate (CH_3_COONa) (Sigma-Aldrich, catalog number: S2889)

7. Dithiothreitol (DTT) (Sigma-Aldrich, catalog number D0632)

8. Ethanol absolute ≥99.8% (VWR, catalog number: 20821.296)

**For plasmid construction:**

1. One Shot^TM^ LB agar plates with antibiotics (Sigma-Aldrich, catalog number: A55802)

2. X-Gal (NZYtech, catalog number: MB025)

3. Dimethylformamide (Sigma-Aldrich, catalog number: 493074)

4. Kit TOPO^TM^ TA Cloning^TM^ (Thermo Fisher Scientific, catalog number: K4575-J10)

5. Maxime^TM^ PCR PreMix i-Taq (iNtRON Biotechnology DR, catalog number: 25025)

6. NZYSpeedy Miniprep (NZYtech, catalog number: MB21001)

**Solutions**

1. Binding buffer (see Recipes)

**Recipes**

**1. Binding buffer**

**Table:** 

Reagent	Stock concentration	Final concentration
NaCl	5 M	2 M
MgCl_2_	1 M	80 mM
Tween-20	20%	0.03%
Tris-HCl, pH 7.5	150 mM	25 mM

Mix all components to prepare 150 μL of a stock solution.

**Laboratory supplies**

1. 10 μL pipette tips (Eppendorf, catalog number: 0030000811)

2. 200 μL pipette tips (Eppendorf, catalog number: 0030000889)

3. 1,000 μL pipette tips (Eppendorf, catalog number: 0030000927)

4. 10 μL pipette filter tips (VWR Avantor, catalog number: 76322-132)

5. 200 μL pipette filter tips (VWR Avantor, catalog number: 76322-150)

6. 1,000 μL pipette filter tips (VWR Avantor, catalog number: 76322-154)

7. Microcentrifuge Eppendorf tubes 3810× 1,5 mL (Eppendorf, catalog number: 0030125215)

8. Real-time PCR tubes, 0.2 mL, in strips (Deltalab, catalog number: 4094.5BP)

9. Erlenmeyer flask 50 mL (Fisher Scientific, catalog number: 10389789)

10. Polycarbonate bottle 1,000 mL (DDBiolab, catalog number: 360516)

11. WHEATON^®^ liquid scintillation 20 mL vial with attached cap (Merck, catalog number: DWK986540-500EA)

12. Petri dish (VWR Avantor, catalog number: 22PS4876)

13. Magnetic stir bar (Sigma Aldrich, catalog number: 41122401)

## Equipment

1. Mastercycler^®^ nexus GX2 thermocycler (Eppendorf, catalog number: 6336000023)

2. QuantStudio^TM^ 5 Real-Time PCR System (Thermo Fisher Scientific, catalog number: A47327)

3. Thermomixer C (Eppendorf, catalog number: 5382000015)

4. NanoDrop 2000 spectrophotometer (Thermo Fisher Scientific, catalog number: ND-2000)

5. Qubit 4 fluorometer (Thermo Fisher Scientific, catalog number: Q33238)

6. Minispin Plus (Eppendorf, catalog number: 5453000015)

7. Magnetic stirrer with heating up to 340 °C (Labbox, catalog number: STIH-020-001)

8. Microcentrifuge 5427 R (Eppendorf, catalog number: 5429000010)

9. E-BOX CX5 (VWR avantor, 733-2832)

10. Wide Mini-Sub Cell GT Horizontal Electrophoresis System, 15 × 7 cm tray, with casting gates (Bio-Rad, catalog number:1704405)

11. KingFisher Flex robot (Thermo Fisher Scientific, catalog number: 5400610)

12. Savant SpeedVac DNA 130 vacuum concentrator (Thermo Fisher Scientific, catalog number: DNA130-230)

13. Telstar Bio II advance plus (Telstar, catalog number: 528287)

14. Telstar V-100 (Telstar, catalog number: 23001)

15. Crop oven, Hareus 7000 series (Vidra Foc, catalog number: TPPP.50042301)

16. Rotator for Eppendorf tubes (Ovan Laboratory equipment, catalog number: 20461000001013)

17. Eppendorf Research plus 10 μL pipette (Eppendorf, catalog number: 3123000020)

18. Eppendorf Research plus 20 μL pipette (Eppendorf, catalog number: 3123000039)

19. Eppendorf Research plus 200 μL pipette (Eppendorf, catalog number: 3123000055)

20. Eppendorf Research plus1000 μL pipette (Eppendorf, catalog number: 3123000063)

21. Racks

22. Liquid nitrogen (N_2_) tank

23. Freezer (-20 °C)

24. Refrigerator (2–8 °C)

## Software and datasets

1. GenBank (https://www.ncbi.nlm.nih.gov/nuccore, 7/June/2024)

2. BLAST (https://blast.ncbi.nlm.nih.gov/Blast.cgi, 7/June/2024)

3. Clustal Omega (https://www.ebi.ac.uk/Tools/msa/clustalo/, 7/June/2024)

4. PrimerExplorer V5 (https://primerexplorer.jp/e/, 10/June/2024)

5. RNAfold WebServer (http://rna.tbi.univie.ac.at/cgi-bin/RNAWebSuite/RNAfold.cgi, 4/July/2024)

6. SnapGene (version 7.1.2, released 28 February 2024); requires a license

7. Design and Analysis Software (version 2.8, released 18 January 2024); requires a license

## Procedure

**A. Sample collection and nucleic acid extraction**

1. Collect oropharyngeal swabs by gently inserting a sterile swab into the trachea through the oral cavity, rotating it carefully against the mucosal surface for several seconds to ensure adequate sampling.

2. Place the swab immediately into an Eppendorf sterile tube containing 1× PBS and keep it refrigerated or on ice until processing in the laboratory.

*Note: Different swabs from the same animal or pools from different animals can be performed by resuspending them in the same Eppendorf sterile tube containing 1× PBS.*

3. Extract nucleic acids from 200 μL of oropharyngeal swab samples using MagMAX Viral/Pathogen I Nucleic Acid Isolation kit with the automated robot KingFisher Flex, following the recommended protocol.

4. Aliquot all purified nucleic acids into 0.2 mL PCR tubes (10 μL per tube) and store them at -80 °C until use.

**B. Amplification using purified nucleic acid templates**

**Figure 1.**
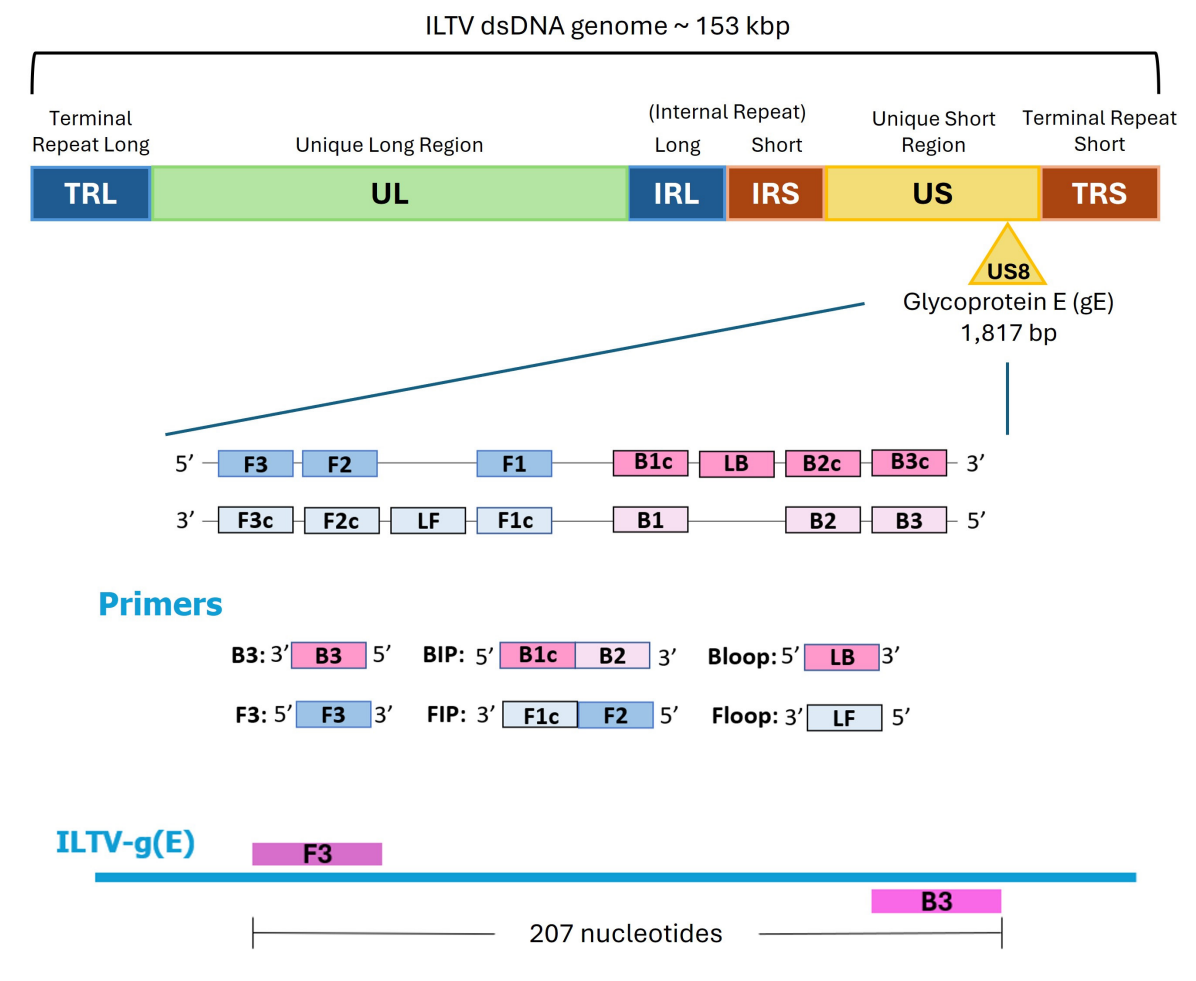
Localization of the six primers required for loop-mediated isothermal amplification (LAMP) amplification of the ILTV *gE* gene. The outer primers are F3 and B3, which are separated by 207 nucleotides. The inner primers are FIP and BIP, each composed of two sequences: FIP consists of F1c (complementary to the F1 region) and F2 (matching the F2 region), while BIP consists of B1c (complementary to B1) and B2 (matching B2). The loop primers are LF (Floop) and LB (Bloop).

1. Loop-mediated isothermal amplification (LAMP) reaction

a. LAMP primers design (Figure 1).

In our work, eight ILTV nucleotide sequences available in GenBank were aligned using Clustal Omega. A highly conserved region within the glycoprotein E (*gE*) gene alignment (nucleotides 693–900) was identified and selected as the target for primer design. The aligned sequences were imported into Primer Explorer V5, using the default automatic judgment parameter settings. The primer set with the highest dG stability (lowest dimerization tendency) was selected as the best candidate.

*Note: Conventional LAMP requires four primers (F3, B3, FIP, and BIP) recognizing six distinct regions of the target sequence, while the inclusion of loop primers (Floop or LF and Bloop or LB) can accelerate amplification by introducing two additional annealing sites [6,9]. Primer design is a critical step, as the large number of primers increases the risk of secondary structure formation and nonspecific amplification. Therefore, primers should be carefully screened for homodimers, heterodimers, and hairpins, and validated experimentally using non-template controls. Established software tools such as PrimerExplorer or NEB LAMP Primer Design are commonly used; however, new software tools are frequently developed to support oligonucleotide design and optimization (e.g., [10]).*

b. Primer mix preparation:

*Note: Work at a lab bench, preferably in a PCR cabinet, after spraying with alcohol. Clean the pipettes with DNA Zap for degradation solution.*

i. Prepare the 100 μM primers by adding autoclaved Milli-Q water according to the manufacturer’s instructions.

ii. Shortly vortex and spin the tubes. Leave 20 min on ice to allow complete resuspension.

iii. Primer sequences for ILTV amplification of gen-*gE* are presented in Table 1.

iv. Dilute the primers at the concentration shown in Table 2.

*Note: Different primer concentrations have been tested for these assays. If primers are changed, new ratios and concentrations should be optimized.*

v. Prepare the primer mix for each primer set (ILTV-*gE*) as shown in Table 3.

**Critical**: Prepare primer mix to avoid cross-contamination.

vi. Aliquot the primer mixes in tubes of 60 μL each.

*Note: Do not reuse the primer mix on different days to avoid cross-contamination.*

vii. Freeze the primer mixes at -20 °C.

c. For LAMP reaction preparation, add the following reagents in a real-time PCR tube (Table 4).

**Critical**: To avoid cross-contamination, it is highly recommended to aliquot the master mix once it is thawed for the first time, adding 12.5 μL into real-time PCR tubes.

i. Briefly vortex and centrifuge the reaction tube. Prepare the reaction out of the ice to allow the uracil-DNA glycosylase (UDG) system to work.

*Note: The UDG system prevents carryover contamination by degrading uracil-containing amplicons from previous reactions, thereby reducing the risk of false-positive results.*

**Figure 2.**
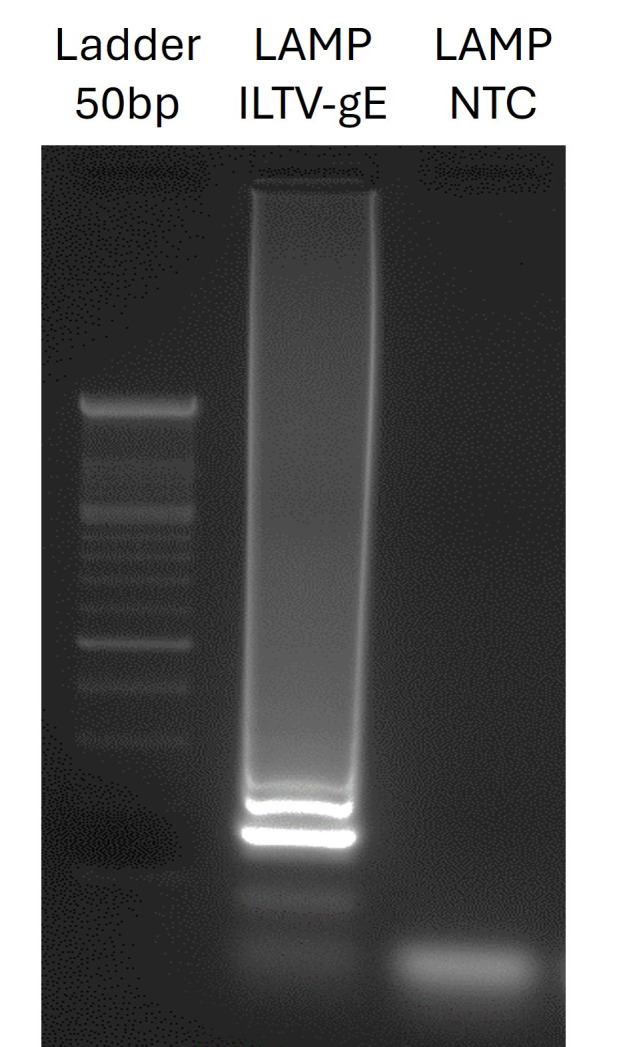
Agarose gel electrophoresis of the loop-mediated isothermal amplification (LAMP) product. Lanes show ladder (molecular weight 50 bp), positive ILTV-*gE* sample, and non-template control (NTC). The positive sample shows the expected band, while no bands are detected in the negative control, confirming the specificity of the assay.

ii. Set up the thermal cycle at 65 °C for 30 min for amplification and subsequent enzyme inactivation at 80 °C for 5 min.

iii. Leave the LAMP product at 4 °C until the electrophoresis.

iv. Prepare an agarose gel at 2% in TAE 1× and GelRed or RedSafe (700 μL of 100× GelRed for 100 mL of gel).

v. Mix 1 μL of 6× DNA loading buffer with 5 μL of LAMP product. Load it into a 2% agarose gel with 560 μL of 100× GelRed. Add 5 μL of 50 bp DNA ladder.

vi. Perform DNA electrophoresis at 100 V for 30 min (Figure 2).

**Table 1.** Primers

**Primer**	**Sequence 5′-3′**	**Tm (**°C)
ILTV-gE-F3	GGCCATGGAAACTACATCGT	62.2
ILTV-gE-B3	GGTCGGTGGGAAGACTRT	60.1–63.1
ILTV-gE-FIP	CCTCTGACGGCTTGATGGTGTCGCGTTCTTTCGCACGTAGA	76.9
ILTV-gE-BIP	CCACGCGCACGTGGAATTACATAGGTGGGGCTGTTGTCG	77
ILTV-gE-Floop	ATCTCCACCTCGTGCGGTG	65.9
ILTV-gE-Bloop	TGCTGCCGTTTCATGAACTCA	64.8

**Table 2.** Primer concentrations

Primer	Initial concentration	Final concentration	Final volume
External primers	100 μM	5 μM	100 μL
Inner primers	100 μM	40 μM	100 μL
Loop primers	100 μM	10 μM	100 μL

**Table 3.** Final primer concentrations n/a, not applicable

	Primer	Initial concentration	Volume
External primers	ILTV-gE-F3	5 μM	100 μL
	ILTV-gE-B3	5 μM	100 μL
Inner primers	ILTV-gE-FIP	40 μM	100 μL
	ILTV-gE-BIP	40 μM	100 μL
Loop primers	ILTV-gE-Floop	10 μM	100 μL
	ILTV-gE-Bloop	10 μM	100 μL
	Total	n/a	600 μL

**Table 4.** LAMP reaction composition

Reagent	Final concentration	Volume
Primer mix	0.16 μM FIP primer 0.16 μM BIP primer 0.1 μM F3 primer 0.1 μM B3 primer 0.2 μM Floop primer 0.2 μM Bloop primer	6 μL
WarmStart master mix	1×	12.5 μL
Milli-Q water	n/a	4 μL
Purified nucleic acid template	n/a	2.5 μL
Total	n/a	25 μL

2. Quantitative loop-mediated isothermal amplification (qLAMP)

*Note: Work at a lab bench, preferably in a PCR cabinet, after spraying with alcohol. Clean the pipettes with DNA Zap for degradation solution.*

a. For primer mix preparation, follow the instructions indicated in Tables 1 and 2.

b. Prepare the following reaction (Table 5) in a PCR tube at room temperature.

c. Briefly vortex and centrifuge the reaction tube and place on ice.

d. Set up the QuantStudio 5 at 65 °C for 40 min with an end-point melt curve step (95 °C for 15 s, 60 °C for 1 min, and 95 °C for 1 s).

*Note: To obtain accurate fluorescence readings, program 40 cycles (one cycle per minute) and record one fluorescence measurement per minute. This setup will produce an amplification curve comparable to that of a PCR reaction.*

**Figure 3.**
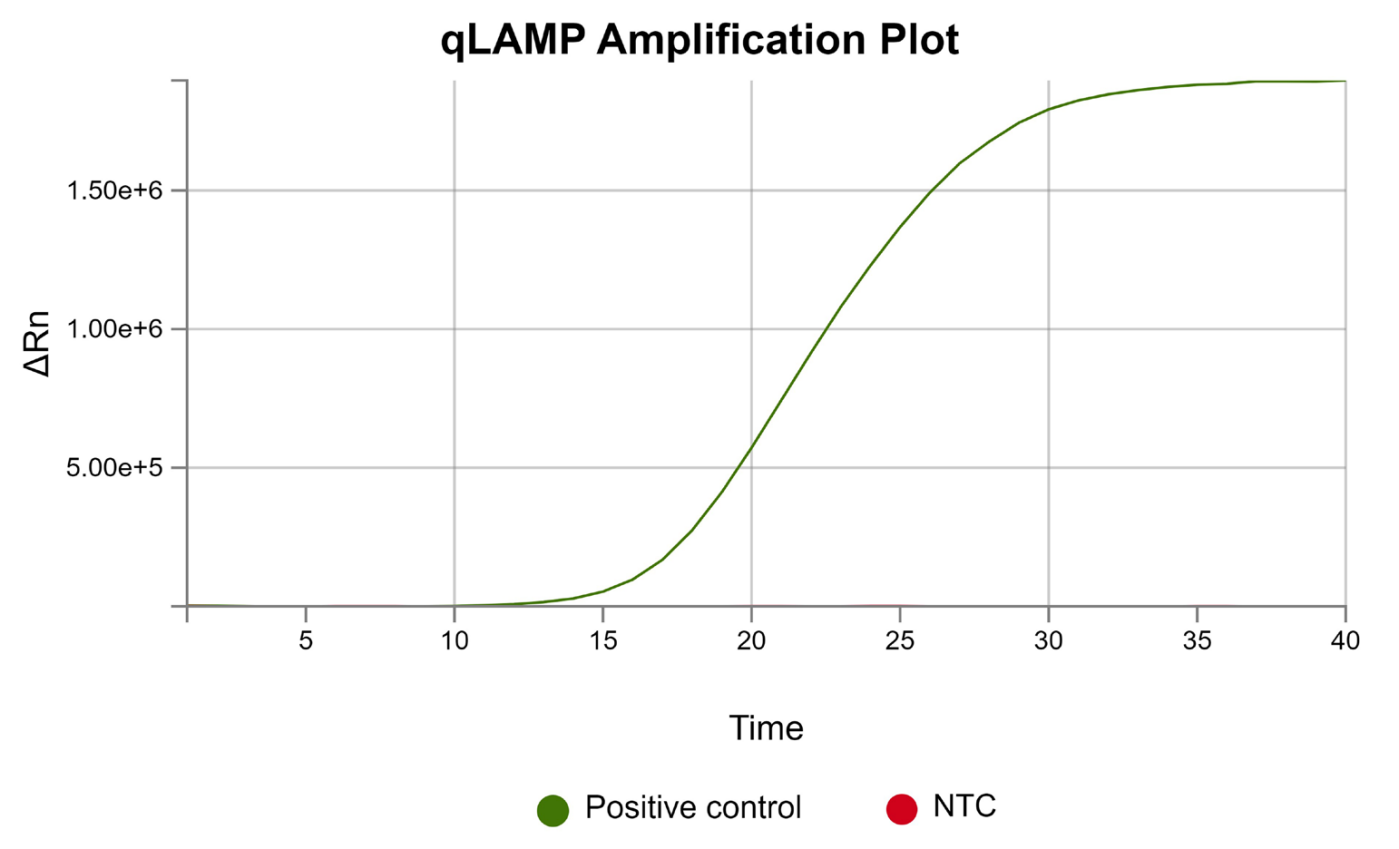
Real-time loop-mediated isothermal amplification (LAMP) fluorescence amplification curves. The positive sample shows a characteristic increase in fluorescence intensity over time, indicating successful amplification. The non-template control (NTC) remains not visible at baseline levels, confirming the absence of nonspecific amplification or contamination.

e. See the results with the Design and Analysis 2.8 software (Figure 3).

**Table 5.** qLAMP reaction composition

Reagent	Final concentration	Volume
Primer mix	0.16 μM (FIP/BIP primers) 0.1 μM (F3/B3 primers) 0.2 μM (loop LF/LB primers)	6 μL
WarmStart master mix	1×	12.5 μL
LAMP fluorescent dye	n/a	0.5 μL
Milli-Q water	n/a	3.5 μL
Purified nucleic acid template	n/a	2.5 μL
Total	n/a	25 μL

**C. Amplification from crude samples**

*Note: Crude samples (respiratory swabs resuspended in 1 mL of PBS) were tested directly without nucleic acid extraction.*

1. Quantitative loop-mediated isothermal amplification (qLAMP)

*Note: Work at a lab bench or in a PCR cabinet after spraying with alcohol. Spray the pipettes with DNAseZap degradation solution.*

a. Thaw the crude samples from -80 °C on ice.

b. For primer mix preparation, follow the instructions indicated in Tables 1 and 2.

c. Prepare the following reaction (Table 6) in a PCR tube at room temperature.

**Table 6.** qLAMP reaction composition.

Reagent	Final concentration	Volume
Primer mix	0.16 μM (FIP/BIP primers) 0.1 μM (F3/B3 primers) 0.2 μM (loop LF/LB primers)	6 μL
WarmStart master mix	1×	12.5 μL
Fluorescent dye	n/a	0.5 μL
Milli-Q water	n/a	1 μL
Crude sample	n/a	5 μL
Total	n/a	25 μL

d. Briefly vortex and centrifuge the reaction tube and place on ice.

e. Set up the QuantStudio 5 at 65 °C for 30 min with an end-point melt curve step (95 °C for 15 s, 60 °C for 1 min, and 95 °C for 1 s).

*Note: To obtain accurate fluorescence readings, program 40 cycles (one cycle per minute) and record one fluorescence measurement per minute. This setup will produce an amplification curve comparable to that of a PCR reaction.*

f. See the results with Design and Analysis 2.0 software.

**D. Synthesis of AuNPs**

1. Synthesis using the Turkevich method [11] with a few modifications:

a. Preheat a hotplate to 100 °C with constant, vigorous stirring.

b. Add 30 mL of MilliQ water and a magnetic stir bar to a 50 mL Erlenmeyer flask. Place the flask on the hotplate and maintain stirring.

c. Once the water reaches boiling, add 40 μL of a 50 mM HAuCl_4_ solution.

d. Immediately add trisodium citrate dihydrate diluted in water to a final concentration of 1%. Allow the mixture to reflux for 10 min.

*Note: Observe the color changes in the mixture as nanoparticles form.*

e. After 10 min, add an additional 40 μL of 50 mM HAuCl_4_ and continue refluxing for another 10 min.

f. Remove the Erlenmeyer flask from the hotplate and allow the suspension to cool to room temperature.

g. To remove the excess of citrate from the synthesis, wash the AuNPs once at 8,000× *g* for 10 min in 1× PBS.

*Note: Due to inherent variability in nanoparticle synthesis, a narrow dynamic size distribution is expected. Therefore, after the centrifugation step, if some nanoparticles are still visible in the supernatant, the sample should not be recentrifuged. Instead, the supernatant should be carefully collected, as this approach selectively reduces the fraction of smaller particles (<20 nm) while retaining the desired population.*

h. Quantify the synthesized AuNPs by acquiring their UV-Vis spectra on a NanoDrop or an equivalent spectrophotometer. Adjust the suspension to reach a maximum absorbance between 518 and 524 nm, with a final absorbance value of 1.5.

*Note: AuNP sizes can be evaluated using electron microscopy or dynamic light scattering. However, high purity and a narrow size distribution are not strictly required for the binding assay.*

i. For storage, keep the nanoparticle solution at 4 °C.

*Note: It is recommended to aliquot the batch in glass vials of 2 mL, as prolonged contact with plastic surfaces may induce aggregation of the AuNPs.*

2. DNA probe design

a. Identify the target DNA sequence using the expected or sequenced amplicon from LAMP amplification.

b. Design a single-stranded oligonucleotide (probe) complementary to the target sequence of 18 nucleotides in length.

*Note: The DNA probe must not hybridize with any of the primers used in the LAMP reaction. Although it is generally advisable to design the probe within the region located between the FIP and BIP primers, this is not strictly required as long as at least an 18-nucleotide complementary region is available elsewhere in the target sequence. Probes of shorter or longer length may also be designed; however, changes in probe length may slightly affect the efficiency of AuNP functionalization.*

Add a 5′ thiol modification (5′-SH-C6 or 5′-SH) to the designed probe during synthesis to enable covalent attachment to the gold nanoparticle surface via strong gold–sulfur chemistry.

c. Order the probe with HPLC purification from a commercial supplier to ensure sequence quality and purity for nanoparticle functionalization.

d. Resuspend the lyophilized probe in sterile, nuclease-free water to a 100 μM concentration and store aliquots of 50 μL at -20 °C until use.

3. DTT reduction of DNA probes

a. Prepare a fresh solution of DTT at 50 mM in 1× PBS, pH 8.0.

b. Mix 50 μL of DNA and 450 μL of DTT in PBS solution.

*Note: You must ensure a ratio of at least 100-fold molar excess of DTT in PBS.*

c. Incubate the mixture at room temperature for 30 min to fully reduce the DNA disulfide bond and activate free thiol groups.

d. Add 1 mL of cold ethanol ~100% at -20 °C.

e. Keep at -20 °C for 2 h.

f. Add 50 μL of sodium acetate 3 M and freeze it overnight at -20 °C.

g. After incubation, centrifuge at 20,000× *g* for 15 min at 4 °C.

h. Remove the supernatant and clean the DNA pellet using 500 μL of ethanol 70%.

i. Centrifuge the mix at 20,000× *g* for 10 min at 4 °C.

j. Remove the supernatant and clean and resuspend the DNA pellet using 500 μL of ethanol 70%.

k. Centrifuge the mix at 20,000× *g* for 8 min at 4 °C.

l. Remove the supernatant and leave the pellet to dry at room temperature.

m. Once the pellet is completely dry, resuspend in 50 μL of MilliQ water.

n. Make calculations for aliquoting the pellet at 50 μM in 1.5 mL Eppendorf tubes and store aliquots of 50 μL at -20 °C until use.

4. AuNP functionalization with DNA probes

a. Incubate 2 mL of 20 nm AuNPs with 80 μL of reduced thiolated probe (50 μM) for 16 h at room temperature in the dark.

*Note: If aggregation of AuNPs takes place during functionalization steps, a more reduced thiolated probe (up to 250 μL) can be added to stabilized nanoparticles.*

b. Divide the sample into two 1 mL aliquots in 1.5 mL Eppendorf tubes.

c. Add 40 μL of 5 M NaCl to each aliquot and incubate for 2 h at room temperature in the dark.

d. Add 0.17 μL of 20% Tween-20 to each microcentrifuge tube.

e. Perform salt aging by concentrating the samples in a SpeedVac at 42 °C for 2–4 h, until the final volume is approximately 250 μL.

f. Add 90 μL of 0.1× PBS and 800 μL of 0.1× PBS supplemented with 0.1 M NaCl to each tube.

g. Centrifuge at 6,700× *g* (10,000 rpm) for 15 min in a minispin Plus.

h. Carefully discard the supernatant and resuspend the pellet in 50 μL of 1× PBS. Take 5 μL for gel analysis. Add 450 μL of 1× PBS to the remaining suspension.

i. Homogenize the suspension using a rotator for Eppendorf tubes at 6,700× *g* (10,000 rpm) for 10 min.

j. Repeat steps D4g–i once.

k. Centrifuge at 4,300× *g* (8,000 rpm) for 5 min.

*Note: Extend centrifugation time if nanoparticles remain in suspension.*

l. Resuspend the pellet in 50 μL of 1× PBS.

*Note: If functionalized AuNPs remain aggregated, perform a heat-shock treatment at 56 °C for 5 min using a thermomixer.*

m. Record the UV-Vis spectra and measure the absorbance at 522 nm using a NanoDrop spectrophotometer. Adjust the sample to an absorbance of around 1.5. Store the DNA-functionalized AuNPs (DNA nanoprobes) at 4 °C until use.

**E. Nanoprobe detection visualization**

1. Visualization method

a. In a 0.2 mL PCR tube, prepare 5 μL of each LAMP amplification product.

*Note: Visualization should be done straight after LAMP amplification is over. If the product is stored at 4 °C, it is recommended to warm it at 65 °C (or LAMP amplification optimal temperature) before binding.*

b. Prepare the binding buffer (see Recipes) in a 1.5 mL Eppendorf tube.

*Note: This binding buffer is highly stable and can be reused for up to one week without any change in visualization. Cover it from the light to maintain Tween-20 activity.*

c. Add 8.5 μL of binding buffer to the end-point LAMP product.

*Note: Prepare negative controls by replacing the LAMP product with a negative LAMP product [amplification of a different target or non-template control (NTC)]. It is important to use a reaction tube due to the presence of salts for amplification.*

d. Pipette 1.5 μL of AuNPs functionalized with DNA nanoprobes in each PCR tube.

**Figure 4.**
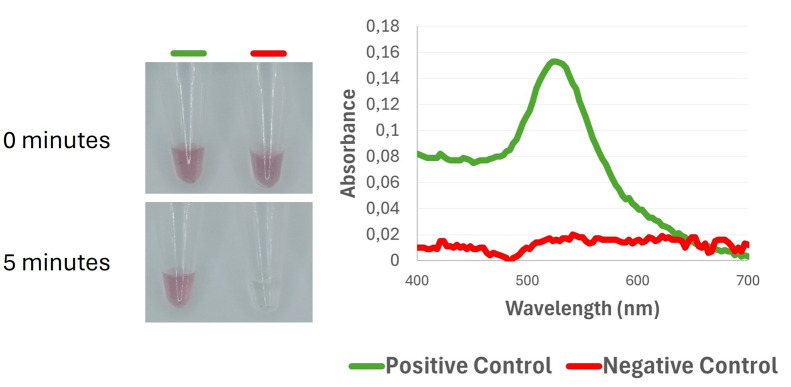
Colorimetric and spectrophotometric discrimination between positive and negative controls using DNA-functionalized gold nanoparticles. (Left) Visual color response of the nanoprobes at 0 min and after 5 min of incubation with the amplification products; the positive control remains in a red color, whereas the negative control shows color fading. (Right) UV-Vis spectra, highlighting the plasmon resonance peak in the positive control (520–540 nm), and its loss in the negative control due to the lack of target DNA binding.

**Critical**: Do not mix the nanoprobes with the end-point LAMP product and binding buffer until the detection starts. Once AuNPs are in contact with the saline buffer, clarification and aggregation will start.

e. Incubate the reaction mixtures at room temperature for 5 min.

f. Inspect the tubes and record absorbance spectra from 400 to 700 nm, focusing on the absorbance maximum at 540 nm (Figure 4).

*Note: For standardization, a plaque and a plate reader can be used with a high number of samples.*

**F. Plasmid construction**

1. Gene fragment amplification for cloning

a. Use external primers designed for LAMP (F3-B3) for PCR amplification.

b. Prepare a 20 μL PCR reaction using Maxime^TM^ PCR PreMix i-Taq (Table 7).

*Note: The kit includes lyophilized mastermix containing enzyme ready in PCR tubes.*

**Table 7.** PCR reaction composition

Reagent	Final concentration	Volume
F3 primer	10 μM	1 μL
B3 primer	10 μM	1 μL
Nuclease-free water	n/a	17 μL
Intended sample for cloning	n/a	1 μL
Total	n/a	20 μL

c. Mix gently and briefly spin down.

d. Run the PCR in a thermal cycler under the conditions shown in Table 8.

**Table 8.** PCR amplification program for the thermocycler

Step	Temperature and time	Cycle
Enzyme activation	95 °C, 2 min	1
Denaturation	95 °C, 15 s	30
Annealing	56 °C, 30 s	
Extension	72 °C, 30 s	
Final extension	72 °C, 5 min	1

2. DNA ligation

a. Set up the reaction as shown in Table 9.

**Table 9.** Ligation reaction components *Provided in the TOPO^TM^ TA Cloning^TM^ kit.

Reagent	Volume
Nuclease-free water	2 μL
PCR product	2 μL
pCR4-TOPO vector*	1 μL
Salt solution*	1 μL
Total	6 μL

b. Incubate in a thermoblock at 37 °C for 10 min.

c. Once your plasmid contains the insert, mix the provided TOP10 competent *E. coli* cells with 6 μL (approximately 10 ng) of plasmid DNA from the previous step. Place the tube on ice for 30 min to allow the DNA to approach the cell membranes.

d. Expose the mixture to a 42 °C heat shock for 1 min in a thermoblock. After 1 min, immediately return the tube to ice.

*Note: After returning to ice, set the tube in a new spot within the ice bucket to ensure it cools quickly and is not affected by heat from other tubes nearby.*

e. Add 250 µL of SOC medium (provided in the TOPO^TM^ TA Cloning kit) to the transformation tube.

f. Incubate at 37 °C for 1 h with shaking at 750 rpm in a thermoblock.

g. Plate 200 µL of the transformation mixture onto LB agar plates with ampicillin and 40 µL of 40 mg/mL X-GAL in dimethylformamide.

h. Incubate overnight at 37 °C.

3. Colony PCR screening

a. Prepare 20 μL PCR reactions with M13 primers and add the components shown in Table 10 using Maxime^TM^ PCR PreMix i-Taq.

**Table 10.** PCR screening reaction

Reagent	Final concentration	Volume
M13-Forward primer	10 pmol/μL	1 μL
M13-Reverse primer	10 pmol/μL	1 μL
Nuclease-free water	n/a	18 μL
Total	n/a	20 μL

b. Pick single colonies using a 200 μL pipette tip and add to the PCR reaction.

*Note: Add at least two negative colonies to ensure correct amplification during the PCR.*

c. Run the PCR in a thermal cycler following the conditions shown in Table 8.

d. Check the presence of the insert in the plasmid by doing an electrophoresis in the same conditions as in LAMP product visualization (section B1c.iv–vi).

e. Perform plasmid extraction using the NZYSpeedy Miniprep kit following manufacturer’s protocol.

4. DNA quantification and detection limit determination

a. Measure plasmid DNA concentration using a Qubit Fluorometer with 1.5 μL of the sample.

b. Store the remaining 40 μL at -80 °C until use.

c. To determine the limit of detection (LOD), prepare 1:5 serial dilutions of purified plasmid DNA.

d. Amplify each dilution using the qLAMP assay, following the conditions described in section A.

## Data analysis

All experimental data were processed and analyzed using Design and Analysis 2 (DA2) and Microsoft Excel. DA2 was employed for initial data handling, including raw data import, baseline correction, and preliminary quality assessment of qLAMP amplification. The software was also used to visualize trends through plots and distribution analyses.

Microsoft Excel was used for subsequent data organization, tabulation, and comparative analysis of UV-Vis absorbance measurements obtained from the NanoDrop. Graphical representations—including line graphs—were generated to summarize the results and facilitate interpretation. All reported values and visualizations were derived from these processed datasets.

## Validation of protocol

All LAMP and qLAMP amplification, binding, and detection experiments were performed in duplicate to ensure reproducibility.

This protocol has been used and validated in the following research article:

- Cea-Callejo et al. [8]. Point-of-Care Diagnostic Test for Rapid Detection of Infectious Laryngotracheitis Virus by Loop-Mediated Isothermal Amplification and Nanoprobes. *International Journal of Molecular Science* (Figures 6 and 7).

## General notes and troubleshooting

All work involving crude clinical specimens positive for ILTV or other avian respiratory viruses should be performed in a biosafety level 2 (BSL-2) cabinet until samples are fully inactivated. Both purified and unpurified respiratory swabs have been shown to be compatible with LAMP-based assays; however, additional specimen types should be validated individually.

To minimize contamination and prevent nucleic acid degradation, all procedures must be carried out using filter tips, fresh gloves, and a strict unidirectional workflow. Clinical samples should be kept on ice whenever possible to preserve RNA integrity. Master mixes and test samples must be handled at separate workstations, each with dedicated pipettes.

Because LAMP assays are highly sensitive to carry-over contamination, particularly in farm environments where viral particles and nucleic acids are widespread, it is strongly recommended to use a LAMP master mix containing a UDG/UNG (uracil-DNA glycosylase) system. This enzymatic safeguard degrades any uracil-containing amplicons from previous reactions, significantly reducing false positives and ensuring assay reliability under field or near-farm conditions.

The LAMP–AuNP nanoprobe system is well-suited for respiratory virus detection from swab samples, which are compatible with established extraction workflows and allow point-of-care application, as validated in this study. In contrast, other specimen types, such as organ tissues, require additional extraction optimization and are less amenable to rapid, on-site testing.
